# Excitation-Wavelength-Dependent Dual Emission in Au_42_ Quantum Rods

**DOI:** 10.1021/acs.nanolett.6c04058

**Published:** 2026-10-12

**Authors:** Guiying He, Lianshun Luo, Mircea Cotlet, Guoxiang Hu, Gangli Wang, Rongchao Jin

**Affiliations:** † Department of Chemistry, 6612 Carnegie Mellon University, Pittsburgh, Pennsylvania 15213, United States; ‡ Center for Functional Nanomaterials, 8099 Brookhaven National Laboratory, Upton, New York 11973, United States; § School of Materials Science and Engineering, 1372 Georgia Institute of Technology, Atlanta, Georgia 30332, United States; ∥ Department of Chemistry, 1373 Georgia State University, Atlanta, Georgia 30302, United States; ¶ Department of Chemistry, The Pennsylvania State University, University Park, Pennsylvania 16802, United States

**Keywords:** gold quantum rods, near-infrared emission, dual emission, anti-Kasha−Vavilov behavior, high-lying intersystem crossing

## Abstract

Dual emissive materials are of particular importance in chemical and biological assays. Herein, we focus on a rod-shaped Au_42_(SCH_2_CH_2_Ph)_32_ nanocluster that exhibits dual emission at 875 and 1040 nm owing to fluorescence (FL) and phosphorescence (PH), respectively. Transient absorption analysis shows a slow intersystem crossing (ISC, ∼500 ps) from lowest singlet S_1_ to triplet T_1_ upon direct S_1_ excitation, leading to dual emission from both S_1_ and T_1_. However, upon excitation of the higher S_*n*_ state, we observe a metastable singlet state (S′). We rationalize two competing decay pathways from S′: (i) internal conversion to S_1_ and (ii) ISC to high triplet T_*n*≥2_. These processes alter the populations of the S_1_ and T_1_ states, which explains the excitation-wavelength-dependent dual emission and FL/PH ratios. The revealed excited-state mechanism and the dual emissive Au_42_ material will serve as a paradigm for future research on atomically precise nanoclusters.

## Graphical abstract

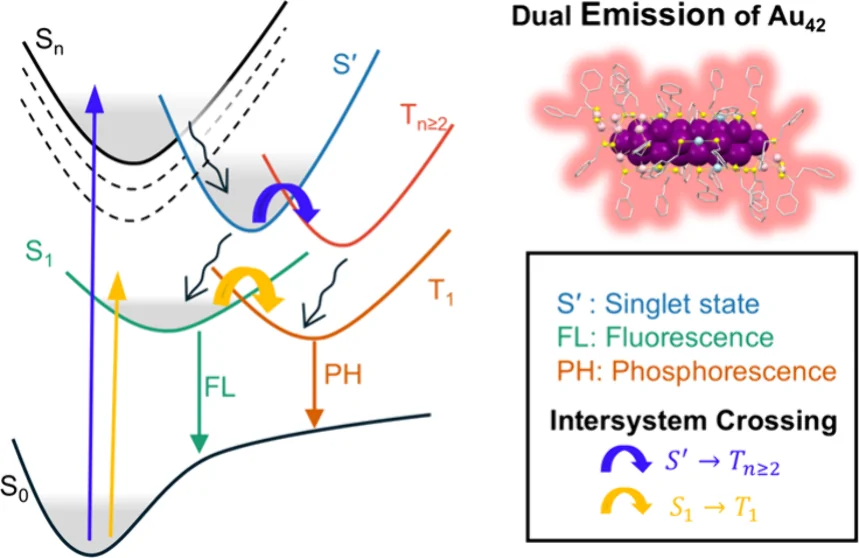

Near-infrared (NIR)-emitting materials are of wide interest because of their potential applications in solar cells,^1–3^ deep-tissue bioimaging and phototherapy,^4–6^ ratiometric sensing,^7^ and even supramolecular quantum encryption.^8,9^ Atomically precise Au nanoclusters (NCs, typically 1–3 nm in diameter), in which the inner Au(0) core is enclosed by exterior Au­(I)-SR “staple” motifs for protection, have emerged as a promising class of NIR emissive materials.^10–14^ In contrast with the metallic-state nanoparticles, whose properties are dominated by surface plasmon resonance (SPR), quantum-sized metal NCs below the size of Au_279_(SR)_84_ (SR = thiolate) exhibit quantized electronic energy levels, and accordingly, multiple discrete absorption peaks, tunable luminescence, and many other molecule-like properties.^15–18^ Through precise control of size, shape, structure, and composition, the photoluminescence of Au NCs can be tuned from the visible to NIR region.^19–22^

According to Kasha’s rule in molecular photophysics,^23^ the emission of photons in a molecular system should come from the lowest excited state (S_1_); thus, a single band results from S_1_ emission (T_1_ in dyes is a dark state) and the emission spectrum should be independent of the excitation wavelengths owing to the fast relaxation to S_1_. A follow-up to Kasha’s rule is that the emission quantum yield (QY) is independent of the excitation wavelengths, which is called the Kasha–Vavilov rule. Although Kasha’s rule applies to the vast majority of the emissive systems, violations are reported in dyes.^24,25^ The prototypical anti-Kasha case is azulene, in which the energy gap between S_2_ and S_1_ is quite large (14 000 cm^–1^), and thus, distinct fluorescence from the S_2_ state was observed.^26^ The fast internal conversion (IC) process from S_1_ to S_0_ in azulene was determined to be 1.9 ps without emission from the S_1_ state.^27–29^ The anti-Kasha effect has received a considerable amount of attention in recent years because of its potential to achieve more efficient photochemistry.^26,30,31^ Due to the fast IC process, the photochemical transformations generally start from the lowest excited S_1_ state, which provides grounds for extending Kasha’s rule to a broader range of excited-state events. The high electronic S_*n*_ and high vibrational S_1_^*m*^ may be involved in diverse photophysics and chemical transformations, including intersystem crossing (ISC),^32,33^ photoisomerization,^34^ and excited-state energy transfer (EET).^35,36^

In contrast, the emission of Au NCs is generally from the S_1_ and T_1_ states owing to the fast relaxation of hot carriers and emissive nature of T_1_,^19–22,37–40^ as opposed to dark T_1_ of organic dyes. Dual emission in Au NCs was previously observed in Au_24_(SR)_20_ with a bitetrahedral kernel structure, but the photoluminescence quantum yield (PLQY) was quite low (∼2%).^41^ Recently, we reported a rod-shaped Au_42_(PET)_32_ nanocluster (PET = SCH_2_CH_2_Ph, hereafter termed Au_42_) with interesting optical properties; for instance, the absorption spectrum of Au_42_ resembles that of bacteriochlorophylls and exhibits dual emission at 875 nm (fluorescence (FL)) and 1040 nm (phosphorescence (PH)) because of the small energy gap (*ΔE* ∼ 0.2 eV) between S_1_ and T_1_.^11,42,43^ The QY of dual emissive Au_42_ is at least 6 times higher than that of Au_24_.^11,41^ The QY of Au_42_ can be further enhanced by embedding them in polystyrene films or by interactions with amide molecules in solution, each of which effectively suppresses the nonradiative process.^42^ It is worth noting that Wu’s group also reported a different synthesis of Au_42_.^43^ The ISC rate was estimated to be 1.6 × 10^9^ s^–1^ by determining a *τ*_FL_ of 0.56 ns and a *Φ*_ISC_ of 0.87 using time-resolved fluorescence and transient absorption measurements.^44^ However, femtosecond transient absorption measurements in the visible region indicate a faster ISC (8.0 × 10^10^ s^–1^).^45^ The broad spectra in the visible region may allow important information to be missed. In theoretical work, computations on the intersystem crossing and radiative/nonradiative decays have been reported.^46,47^ These experimental and theoretical results imply quite intriguing excited-state dynamics in Au_42_ and are largely different from the previously reported NCs;^45,47–52^ thus, a detailed study is required to understand the interplay of excited states.

Here, we report the intricate excited-state dynamics in Au_42_ (its structure is shown in Figure 1a) by performing systematic spectroscopic probing to determine the QY and exciton dynamics at different excitation wavelengths. The steady-state absorption spectrum shows two primary peaks corresponding to the S_*n*_ state (375 nm, 3.31 eV) and S_1_ (806 nm, 1.54 eV), with additional relatively weak broad absorption between these two states. Interestingly, the ratio of FL and PH emission of Au_42_ is dependent on the excitation wavelength. The PH decreases from 10.4% (excitation at 380 nm) to 8.7% (excitation at 810 nm). Transient absorption measurements of Au_42_ from 810 to 1550 nm are further performed to reveal the mechanism of excitation-wavelength-dependent exciton dynamics. The current results indicate fast (8.6 × 10^10^ s^–1^) and slow (1.1 × 10^9^ s^–1^) intersystem crossing (ISC). A high-energy metastable singlet state (S′) is observed in transient absorption spectra upon higher-energy excitation (>1.75 eV), which decays in ∼3.5 ps, including the internal conversion (IC) process to S_1_ and the ISC process to triplet state T_*n*≥2_. It is exciting that the high singlet state can be obtained with an excitation energy of 1.75 eV, which is much lower than the S_*n*_ energy (3.31 eV) from the absorption spectrum. Overall, the observed anti-Kasha–Vavilov behavior leads to the triplet-state formation from both the high singlet state (S′) and the S_1_ state via two ISC processes.

**Figure 1:**
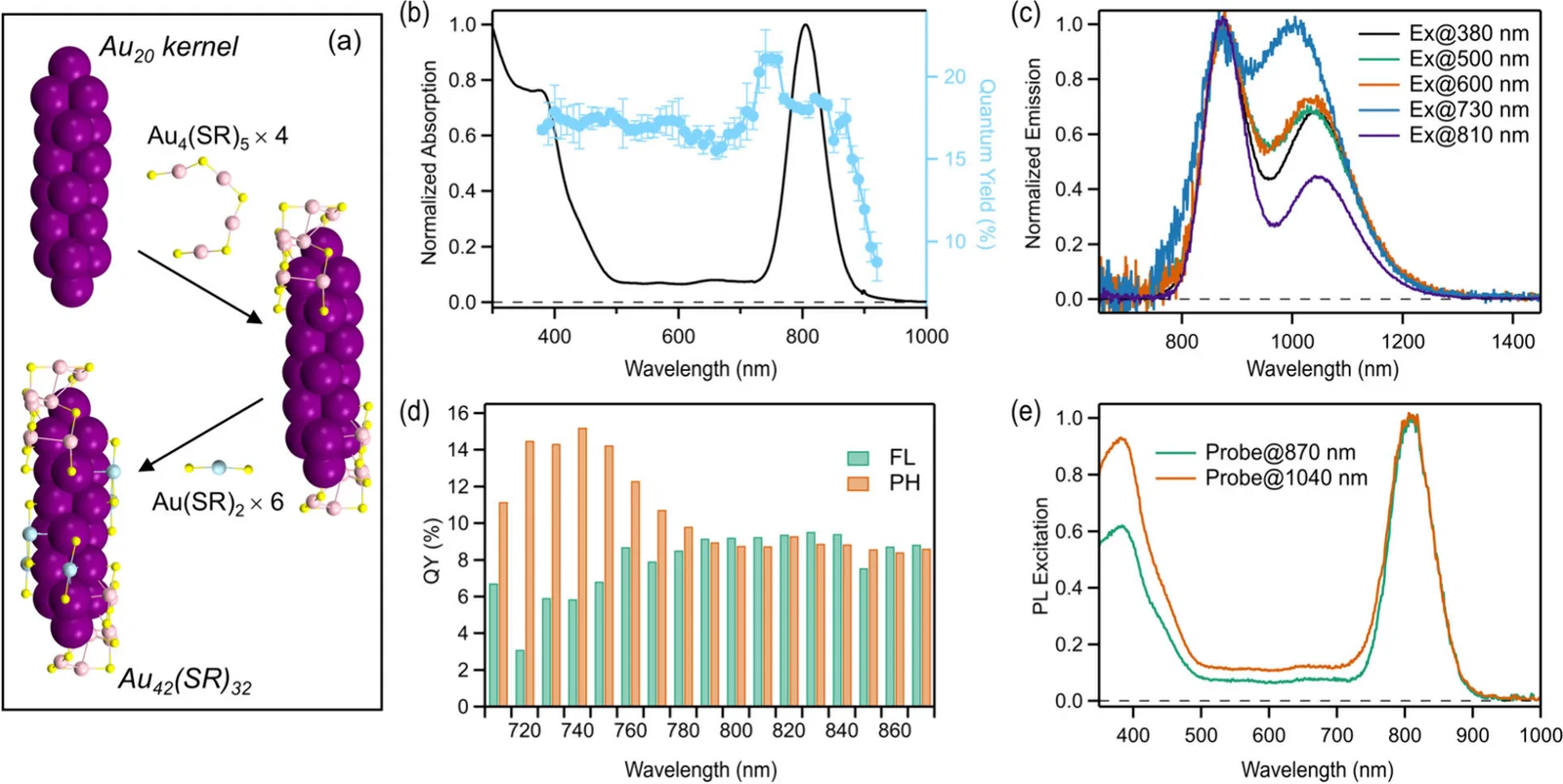
(a) X-ray structure of Au_42_. (b) Steady-state absorption spectrum along with the excitation-wavelength-dependent PLQY. (c) Photoluminescence spectra. (d) Wavelength-dependent quantum yields of FL and PH. (e) Photoluminescence excitation spectra for emission at 870 and 1040 nm.

The Au_42_ quantum rod was synthesized using a method of N-heterocyclic carbene (NHC)-mediated kinetic control reported by our group.^11,49^ Au_42_(PET)_32_ was separated by thin layer chromatography (TLC) (Figure S1), and electrospray ionization mass spectrometry (ESI-MS) was performed to confirm its purity (Figure S2). The Au_42_ absorption spectrum exhibits two strong peaks at 375 and 806 nm with weak absorptions between those peaks (Figure 1b, black curve). Density functional theory (DFT) calculations revealed that the 806 nm peak (S_0_ → S_1_) arises from the HOMO–LUMO transition and the 375 nm peak (S_0_ → S_*n*_) from HOMO–1 to LUMO+2 and HOMO–2 to LUMO+1.^49,53^ The 375 nm absorption band is insensitive to the AR of the rods, which is a common absorption peak as in other Au rods.^49,54–56^ Dual emission at 875 and 1040 nm was observed upon excitation at different wavelengths (Figure 1b,c), which is consistent with our previous measurements in DCM and recent work in toluene.^11,44^ The fluorescence (FL, S_1_ → S_0_) is at 875 nm, and its lifetime is ∼0.72 ns upon excitation at 380 nm in DCM and 0.56 ns in toluene upon excitation at 805 nm. The 1040 nm emission is phosphorescence (PH, T_1_ → S_0_), indicated by its long lifetime of up to 3 μs and also triplet exciton transfer to various triplet acceptors (such as O_2_ and dyes).^44,57–59^

To our surprise, the FL/PH ratio and total PLQY are dependent on the excitation wavelength. Upon excitation at 380–700 nm, the PLQY remains constant (∼17%), but it starts to increase, reaches the highest PLQY (21%) upon excitation of ∼750 nm, and finally decreases when excited by the red side of the S_1_ absorption peak since it is beyond the absorption range (Figure 1b, cyan). In particular, the emission spectra (normalized to the peak of FL) show different ratios of FL to PH (Figure 1c) upon excitation at different wavelengths. The PH yield is relatively constant upon excitation at 380, 500, and 600 nm, while a higher yield is seen upon excitation at 730 nm; the lowest PH yield is observed upon excitation at 810 nm, i.e., direct S_1_ excitation (Figure S3).

The quantum yields of FL and PH are extracted by emission peak deconvolution (Figure 1d). The FL quantum yield is ∼6.4%, while the PH yield is ∼10.4% (upon excitation at 380 nm), which are similar to those upon excitation at 500 and 600 nm. However, the FL yield increases to 9.3% and the PH yield decreases to 8.7% upon excitation at 810 nm. The excitation-wavelength-dependent FL/PH QYs indicate that the normal ISC process from the S_1_ state is not the only channel to generate the triplet state. We speculate that there should be an additional ISC process, which could be a transition between higher singlet and triplet states. Furthermore, the normalized photoluminescence excitation spectrum (PLE) for the 1040 nm emission (PH) is stronger than the PLE for the 875 nm emission (FL) at the short-wavelength (< 760 nm) portion (Figure 1e), indicating that some PH was from higher-energy excitation; in other words, there should be ISC from a high singlet state to a triplet state. In addition, the high energy levels between S_*n*_ and S_1_ should also contribute to PH.

To investigate the intricate dynamics, we performed pump-wavelength-dependent transient absorption (TA) measurements to track the exciton dynamics of Au_42_. We selected a series of excitation wavelengths, including direct S_1_ excitation (810 nm), direct S*_n_* excitation (380 nm), and several intermediate wavelengths (500, 600, and 730 nm).

TA spectra upon direct S_1_ excitation (Figure 2a) exhibit ground-state bleaching (GSB, negative) signals at <820 nm and broad excited-state absorption (ESA, positive) beyond 820 nm. With a short delay (<1 ns), the transient spectra consist of a main ESA peak located at 910 nm generated immediately upon excitation, which should be attributed to the S_1_ state. There is a simultaneous increase in broad ESA ranging from 900 to 1200 nm with the decay of the S_1_ state. The final state has a lifetime of 3.1 μs, which can be attributed to a triplet state.^11,42,49,57^ The kinetics of 920 and 1070 nm are shown in Figure 2b to display the decay of S_1_ and concurrent growth of T_1_. Here, the triplet state was generated in 561 ps, which is comparable to the fast fluorescence process (0.56 ns in toluene).^44^ The physical origin of the ISC process was investigated by DFT and time-dependent density functional theory (TD-DFT) methods.^46,47^ The similarity between the geometric structures of S_1_ and T_1_ benefits the ISC process from S_1_ to T_1_ due to the small reorganization energy, while the similarity of the S_1_ and T_1_ electronic structures gives rise to a comparably small spin–orbit coupling (SOC). Thus, the relatively slow ISC rate leads to both S_1_ and T_1_ populations for the dual emission observed. Furthermore, a large energy gap between T_1_ and T_2_ was calculated based on the geometry of the singlet or triplet; thus, the energy flow from S_1_ to T_2_ can be excluded.^46^

**Figure 2:**
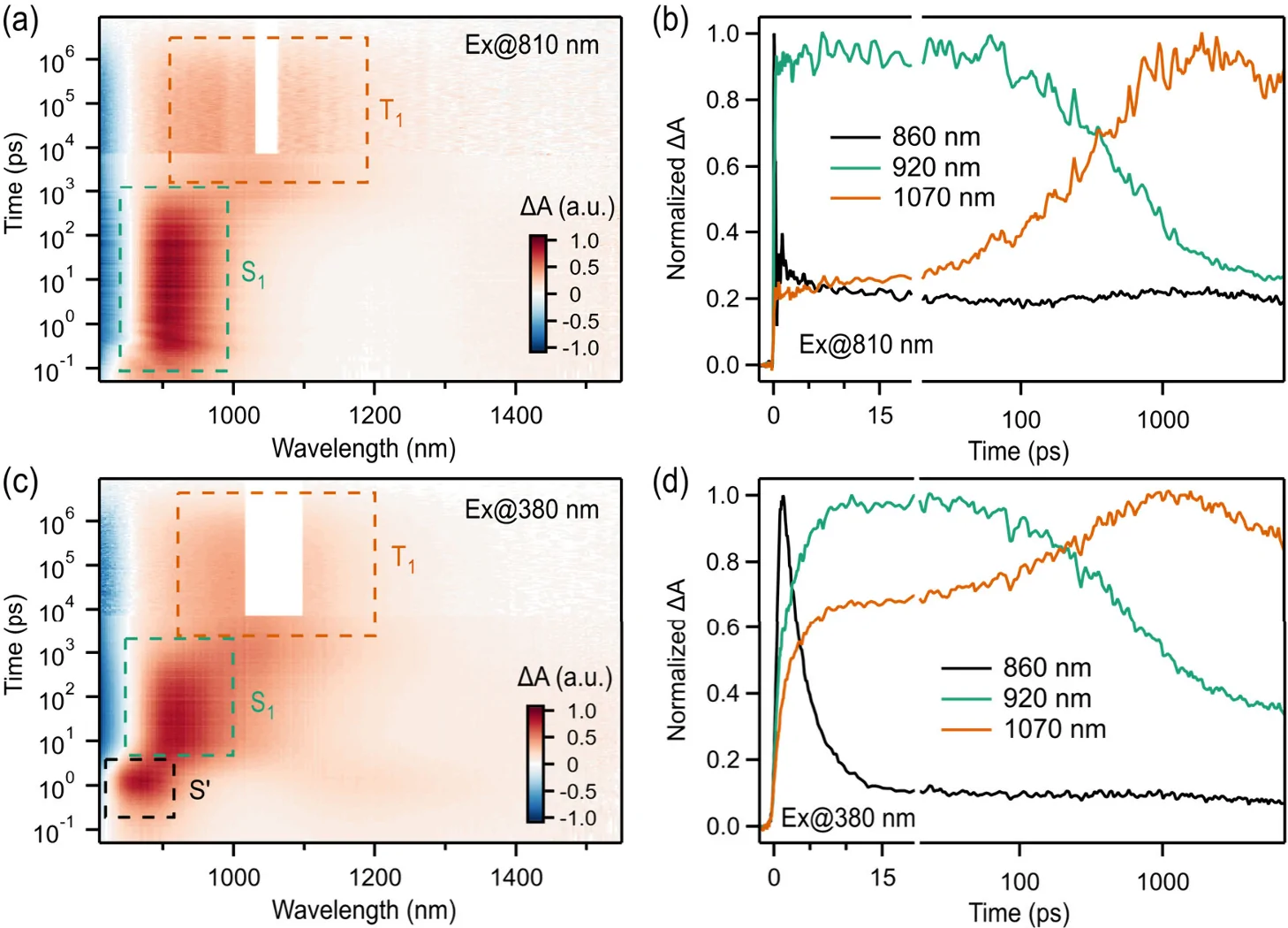
Transient absorption spectroscopy analysis and kinetic cuts at selected wavelengths of Au_42_ excited at (a and b) 810 nm and (c and d) 380 nm. The white boxes for ns-TA in panels a and c denote the removal of the residual pump-laser peak of the probe.

In comparison, the TA data map upon S*_n_* excitation (380 nm) is shown in Figure 2c. As opposed to the S_1_ excitation, there is an obvious ESA peak located at 860 nm, which decays in 3.4 ps with the simultaneous rise of the S_1_ and T_1_ states (Figure 2d, black decay vs green and orange growth). Similar to other rods,^56^ we identified that fast ISC occurs between S′ and the high triplet state (T_*n*≥2_). Because of the fast internal conversion between T_*n*_ and T_1_, we cannot resolve the signals of T_*n*_. The fast rise of the triplet state in the first few picoseconds supports our assumption. Similar dynamics were observed in the visible region probe TA measurements (Figure S6). Considering the overlap of excited species, there are distinguished ESA peaks for individual species: 490 nm for S′, 610 nm for the S_1_ state, and 550 nm for the triplet state.^45^

From the TA data upon excitation at 380 nm, we rationalize that a fast ISC process contributes to the triplet population via the high triplet state. The metastable singlet state (S′) can still be observed upon excitation at 500 or 600 nm (Figure 3a and Figures S7–S9), which possesses a much smaller oscillator strength; hence, weak absorption was observed in the steady-state spectrum. This relaxed state is higher than the emissive S_1_ state, which should be in resonance with a high triplet state (T_*n*≥2_), hence, fast intersystem crossing. We include the S′ state when setting the target for excitation at 500 and 600 nm as in the case of 380 nm excitation. The transient absorption results upon excitation at 600 nm (Figure 3a) show an obvious initial excited state located at 860 nm, which is similar to the TA data upon excitation at 380 nm. Even though there is no obvious S′-state signal in TA data upon excitation at 730 nm (Figures S10 and S11), we observe a red-shift at a short delay time, in which we assigned a minor S′ population to the triplet state.

**Figure 3:**
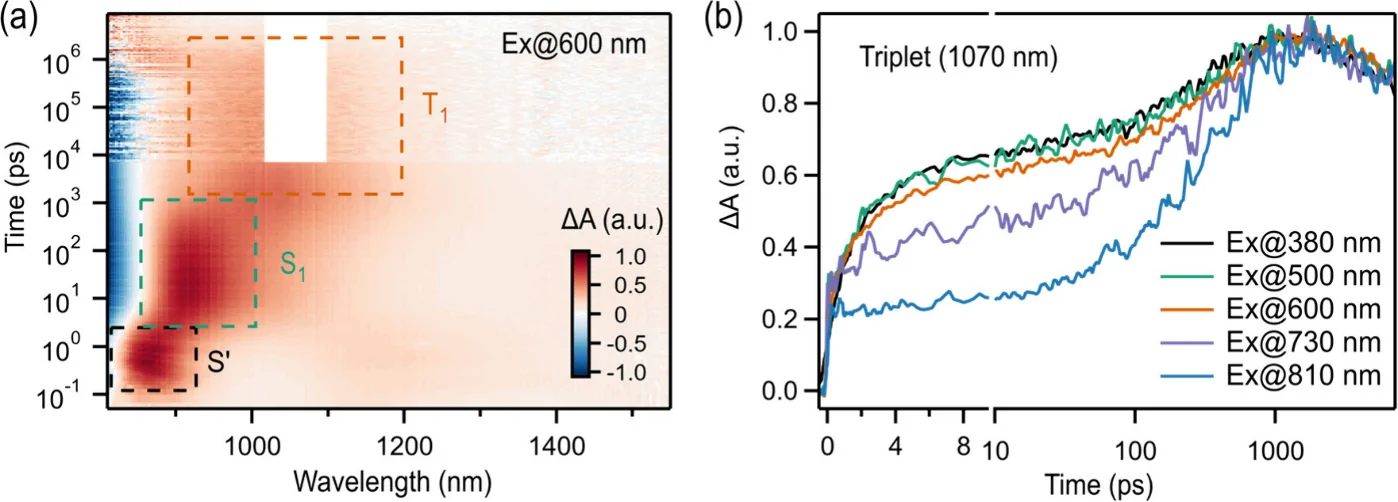
(a) Transient absorption spectroscopy of Au_42_ at 600 nm excitation in toluene and (b) kinetic cuts of 1070 nm from the TA data for all of the excitation wavelengths. The white boxes for ns-TA in panel a denote the removal of the residual pump-laser peak of the probe.

The kinetics at 1070 nm were extracted from the wavelength-dependent TA data to demonstrate the channels of triplet formation (Figure 3b). A fast rise of triplet formation was observed in the first 10 ps for excitation at 380, 500, and 600 nm, which is absent for excitation at 810 nm. There is a subtle rise for excitation at 730 nm, which suggests that the energy of the excited state (S′) may be slightly higher than 1.7 eV (i.e., 730 nm). We note that the slow ISC process (the one with hundreds of picoseconds) applies to TA data of all excitation wavelengths, while fast ISC is for excitation at only ≤730 nm.

As per the above description, triplet formation occurs in less than 500 ps and the lifetime is up to 3 μs. The dominant excited species should be the triplet state at a time delay of 7 ns (maximum delay of fs-TA). As shown in Figure 4a, the kinetics of the bleaching signal (∼810 nm) indicate the exciton population, in which the residual of GSB at 7 ns indicates the population of the triplet state. There is no serious overlap between the GSB and ESA of S_1_ (also between the GSB and ESA of T_1_), since there is no shift in GSB during the normal ISC process (Figures 2 and 5). The triplet yield upon S_1_ excitation is determined to be 62%, which is slightly lower than that of S_*n*_ excitation (70%), indicating the additional ISC process upon S*_n_* excitation.

**Figure 4:**
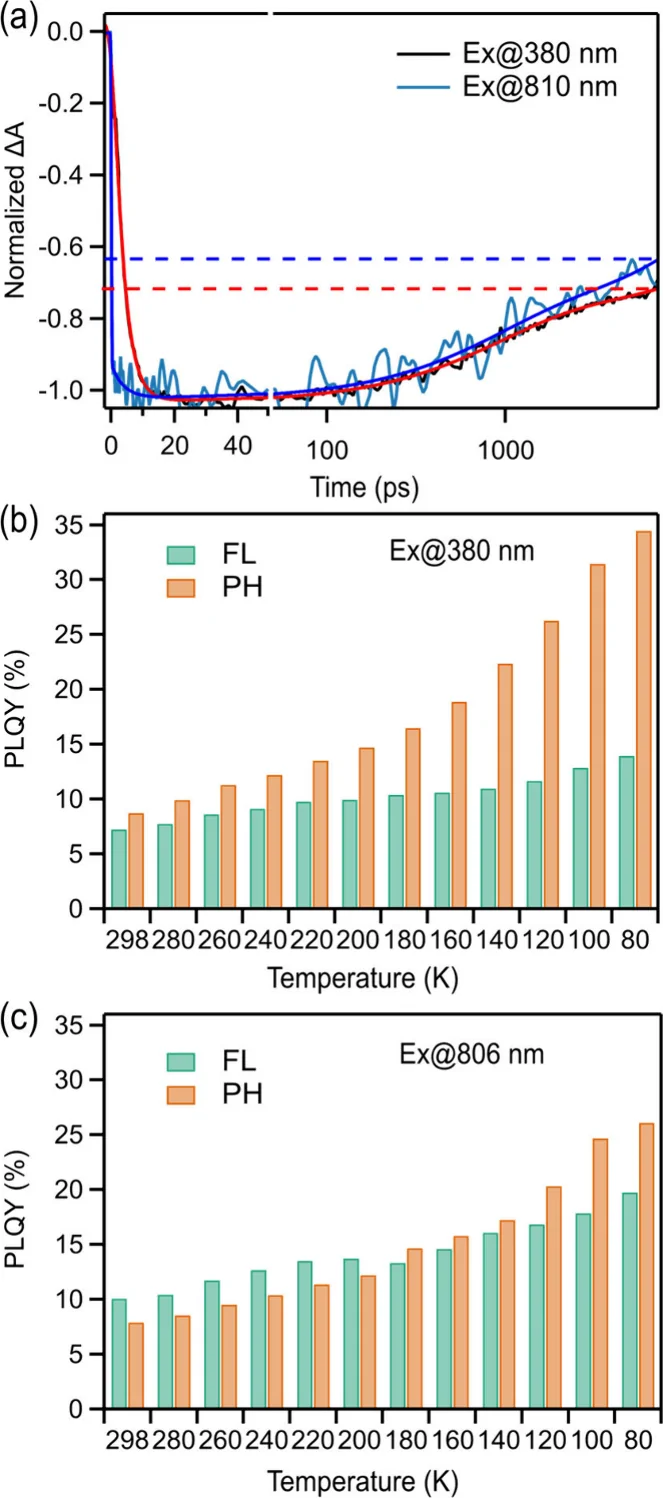
(a) Bleaching signals (∼810 nm) extracted from fs-TA with different excitation wavelengths. (b and c) Temperature-dependent FL and PH QYs of Au_42_ for excitation at 380 and 806 nm, respectively.

**Figure 5:**
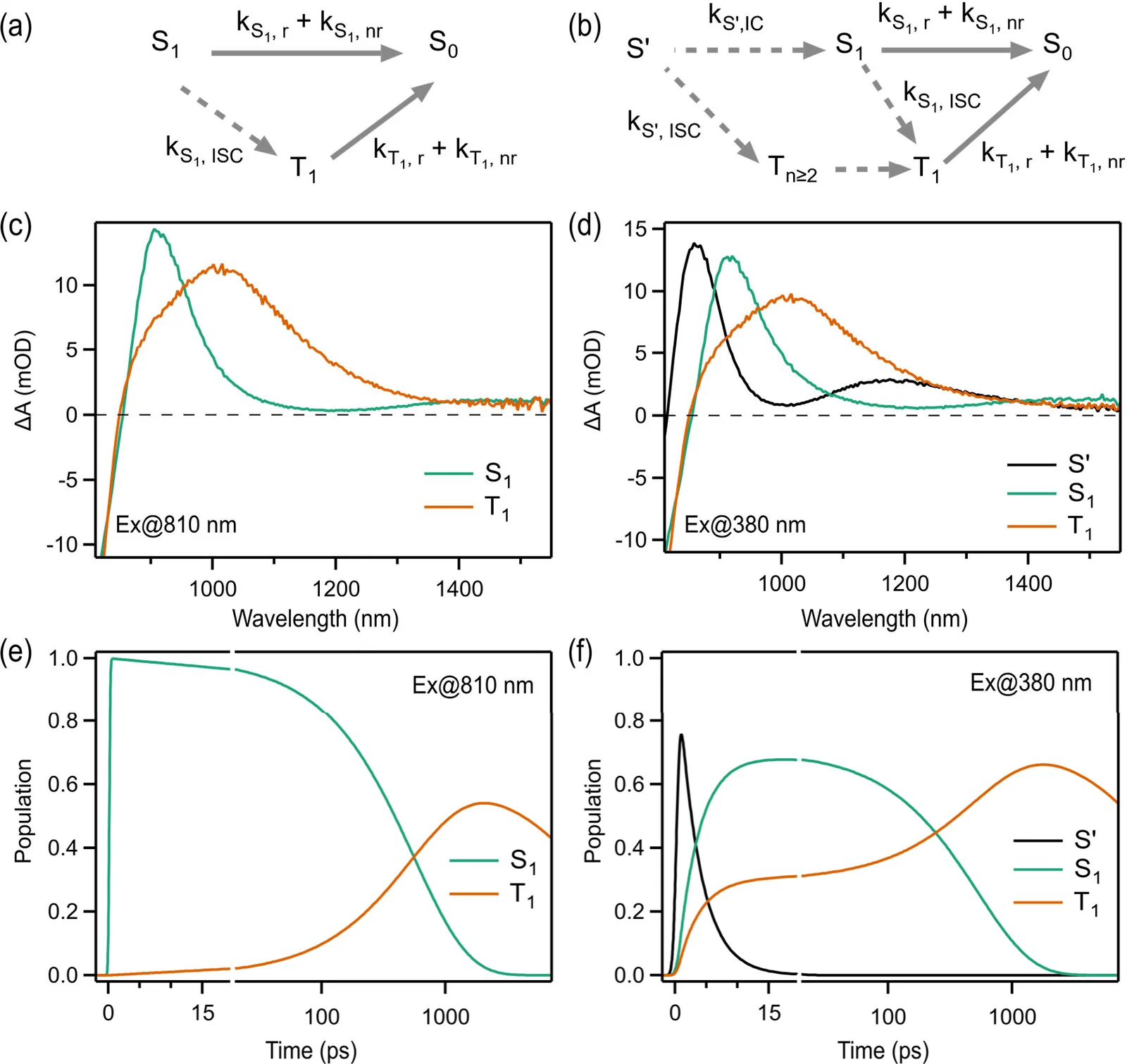
Target analysis based on a branched model for Au_42_ at excitation of 810 and 380 nm. Following the target models (a and b), the species-associated spectra (c and d) and population evolution (e and f) are extracted.

Furthermore, we performed temperature-dependent PL measurements (in Me-THF, from room temperature to 80 K) to determine the singlet and triplet populations by suppressing the effect of the nonradiative process (Table S1 and Figure 4b,c). We assume that the nonradiative process of S_1_ and T_1_ should be similar at 80 K. Even the PLQY can not reach unity at 80 K (only 45.9% upon S_1_ excitation); the relative value of FLQY to PHQY can be used to present the relative ratio of S_1_ → S_0_ to T_1_ → S_0_. It should be noted that the FLQY is ∼19.8% and the PHQY is 26.1%, which indicates that 57% (relative) of the quantum yield is attributed to T_1_ → S_0_, which is consistent with the triplet yield from fs-TA data. Thus, we fix the ratio of S_1_ → S_0_ and S_1_ → T_1_ process from the S_1_ state as 4:6. Upon S*_n_* excitation at 80 K, the relative value of PHQY increases to 71%, indicating an additional triplet formation route.

Based on the above analysis, we use the target model to fit TA data. Upon S_1_ excitation, Figure 5c shows the species-associated spectra (SAS) of S_1_ and T_1_ with the model shown in Figure 5a, whose peaks are located at 910 and 1020 nm, respectively. The normalized population decay shows the triplet rise at 561 ps, which is close to 0.6 (Figure 5e). In contrast, the target model for the case of S_*n*_ excitation (Figure 5b) is more complicated, in which the fast relaxation of the excited state was extracted (860 nm), including the fast IC process (S′ → S_1_) and ISC process (S′ → T_*n*≥2_). We set the S′ → S_1_ path as 0.7, while the fast ISC process is 0.3. Ideally, the PH ratio can reach 0.72 (i.e., 0.3 + 0.7 × 0.6), which is consistent with the PH increasing upon high-energy excitation. The SAS (Figure 5d) shows the same S_1_ and T_1_ spectra with an additional S′ spectrum. Thus, we observe a fast and then slow rise of the triplet state from population evolution (Figure 5f).

The fitting results based on the branched model (Scheme 1) for the TA data upon excitation with different wavelengths are summarized in Table 1. The proposed excited-state evolution mechanism includes the vertically excited S_1_ or S_*n*_ state, and the follow-up excited state of each (S′ or T, respectively). Besides the above description for excitation by 380 and 810 nm, selected excitation between the S_*n*_ and S_1_ peaks (e.g., 500, 600, and 730 nm) and the band edge excitation at 900 nm are shown (Figures S12 and S13). For 500 and 600 nm excitation, the dynamics are similar to those of S_*n*_ excitation, indicating a fast relaxation of S′ and a slow decay of S_1_. The relative ratios of FL and PH are similar, too. As discussed above, there should be an intermediate metastable state (S′), whose energy is close to 1.77 eV (700 nm), which is much lower than the S_*n*_ state (3.26 eV, 380 nm) and higher than the emissive S_1_ state (1.42 eV, 875 nm). It should be noted that there are no additional excited states beyond the S′ state. After the initial fast relaxation (∼3.5 ps), the transient absorption results indicate identical signatures of the S_1_ and T_1_ states (Figure S14). Furthermore, the FL decreases and PH increases obviously from 700 to 750 nm (Figure 1d), indicating a favorable transition from the singlet to triplet state. We rule out the direct excitation of triplet states since we did not observe instantaneous formation of triplet states. Since there is no obvious signature of the S′ state upon excitation at 730 nm, we suggest that the high vibronic state of S_1_ should be in resonance with the triplet state, which gives a somewhat accelerated ISC process (464 ps for excitation at 730 nm (Table 1)). The PH decreases then upon excitation at >750 nm, i.e., off-resonance. Band edge excitation occurs at 900 nm, which is slightly lower in energy than the emissive singlet state (Figures S12 and S13). The quantum yield decreases due to less emission from S_1_. The ISC process is also somewhat slower (590 ps (Table 1)) than high-energy excitation.

**Scheme 1:**
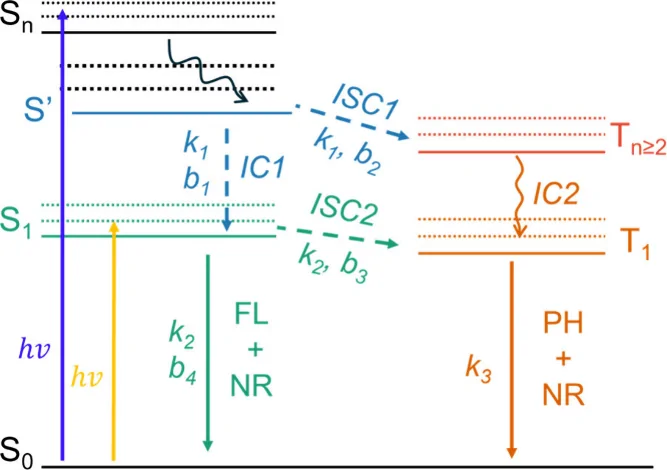
Proposed Excited-State Dynamics of Au_42_ for Different Excitation Wavelengths^a^

a The target model with processes and ratios is used to extract dynamics (details in Table 1).

**Table 1:** Dynamics Parameters (time constants and fractions of excited states) Determined by Targeted Global Fitting of fs-TA Results

	S′ → S_1_(*b*_1_)/T_*n*≥2_(*b*_2_)			S_1_ → T_1_(*b*_3_)/S_0_(*b*_4_)			T_1_ → S_0_
Au_42_	τ_1_ (ps)	*b*_1_	*b*_2_	τ_2_ (ps)	*b*_3_	*b*_4_	τ_3_ (μs)
Ex@380 nm	3.4	0.7	0.3	532	0.6	0.4	2.6
Ex@500 nm	3.9	0.7	0.3	525	0.6	0.4	2.7
Ex@600 nm	3.4	0.7	0.3	521	0.6	0.4	3.1
Ex@730 nm	3.7	0.95	0.05	464	0.6	0.4	3.1
Ex@810 nm	–	–	–	561	0.6	0.4	3.1
Ex@900 nm	–	–	–	590	0.4	0.6	3.0

The dynamic mechanism can be elucidated by the following equations

$$k_{S^{\prime }}=k_{S^{\prime },\mathrm{IC}}+k_{S^{\prime },\mathrm{ISC}}$$

$$k_{S_{1}}=k_{S_{1},r}+k_{S_{1},\mathrm{ISC}}+k_{S_{1},\mathrm{nr}}$$

$$k_{T_{1}}=k_{T_{1},r}+k_{T_{1},\mathrm{nr}}$$

Combining PLQY and TA data, we extracted the rate constants of each decay path. Considering the fast decay of S′, we ignore the nonradiative process of the S′ state. The lifetime of S′ is ∼3.5 ps (*k*_S′_ = 2.9 × 10^11^ s^–1^). The fraction of IC was designated as *b*_1_ (value of 0.7) and the ISC process as *b*_2_ (0.3). Thus, *k*_S′,IC_ = 2.0 × 10^11^ s^–1^ and *k*_S′,ISC_ = 8.6 × 10^10^ s^–1^. The high-lying ISC, like a “skybridge”, leads to fast triplet formation on the order of 10^10^ s^–1^. Then, the PLQY with excitation of 810 nm (FLQY = 9.3%, and PHQY= 8.7%) was used to extract the relative fraction of each process. With the normalized initial population of the S_1_ state, the fraction of *b*_3_ (0.6) should be the slow ISC process while the fraction of *b*_4_ (0.4) can be decomposed as radiative (0.09) and nonradiative (0.31) processes. The lifetime of S_1_ is 561 ps (*k*_S_1__ = 1.8 × 10^9^ s^–1^). Thus, the radiative rate (*k*_s_1_,r_ = 1.7 × 10^8^ s^–1^) and nonradiative rate (*k*_S_1_,nr_ = 5.5 × 10^8^ s^–1^) were determined. Meanwhile, the normal ISC process was determined as *k*_S_1_,ISC_ = 1.1 × 10^9^ s^–1^, which is 2 orders of magnitude slower than the fast ISC from S′. However, relative to the other process, the triplet yield from S_1_ is much higher than that of the longer rods.^56^ Similarly, the decay of the triplet state also includes radiative (0.09) and nonradiative (0.51) processes. The relative fraction of each process was used to determine the rates. The radiative and nonradiative rates of T_1_ are determined as *k*_T,r_ = 5.0 × 10^4^ s^–1^ and *k*_T,nr_ = 2.8 × 10^5^ s^–1^, respectively. We found that both S_1_ and T_1_ are efficient emissive states because of the relatively fast radiative rate constants compared to the corresponding nonradiative rate. Compared to the longer rods, the fast (10^10^–10^11^ s^–1^) ISC process was determined, which should contribute to the high triplet yield. With the increasing length of rods, the rates of nonradiative processes from S_1_ and T_1_ increase drastically, which become the most competitive decay pathways relative to the normal ISC process of S_1_ and emission of S_1_ or T_1_. The fraction of the nonradiative process of S_1_ for Au_42_ is only 31%, whereas this value is much higher for longer rods (69% for Au_60_, 79% for Au_78_, 98% for Au_96_, and 99% for Au_114_). Thus, the normal ISC (S_1_ → T_1_) is seriously suppressed by the accelerated nonradiative process. Similarly, PH was suppressed by the faster nonradiative process of T_1_ for the longer rods, in which we did not observe PH upon S_1_ excitation.^56^

In summary, we have revealed the intriguing excitation-wavelength-dependent PLQY and excited-state dynamics of Au_42_. TA spectra upon direct S_1_ excitation show no trace of higher singlet state. The ISC process occurs from the initially populated S_1_ state. Upon excitation at wavelengths shorter than 700 nm, the additional fast ISC process promotes triplet-state formation, and the TA spectra indicate a distinct S′ state, even though the excitation energy is much lower than that of the S_*n*_ state from the absorption spectrum. Thus, the high-energy singlet state S′ is a relaxed state from the initial photoexcited state with a photon energy larger than 1.7 eV. The lifetime of this hot exciton (S′) is around ∼3.5 ps, whose decay includes IC (S′ → S_1_) and ISC (S′ → T_*n*≥2_) processes. A fast ISC process occurs upon excitation of this state, which results in a higher PH quantum yield. Overall, this work presents the first case of intricate excited-state dynamics in atomically precise nanoclusters. It sets a paradigm for future research on dual emissive nanoclusters and will also promote their applications in near-infrared LED, bioimaging, and solar energy conversion.

## Supplementary Material



## Notes

Methods, details of synthesis, steady-state and time-resolved spectroscopic analyses, Figures S1–S14, and Table S1 (PDF)
