# Supplementary material for: Excitation-Wavelength-Dependent Dual Emission in Au42 Quantum Rods
Source: Nano Lett. 2026 Oct 12;26(41):14409–17. doi: 10.1021/acs.nanolett.6c04058 (PMC13693494; doi:10.1021/acs.nanolett.6c04058)
Supplement: Supplementary file 1 [file nl6c04058_si_001.pdf]

Excitation-Wavelength-Dependent Dual Emission in Au_{42} Quantum Rods

Guiying He,[1,#*] Lianshun Luo,[1,#] Mircea Cotlet,[2] Guoxiang Hu,[3] Gangli Wang,[4] and Rongchao Jin[1,*]

[1]Department of Chemistry, Carnegie Mellon University, Pittsburgh, Pennsylvania 15213, USA

[2]Center for Functional Nanomaterials, Brookhaven National Laboratory, Upton, NY, 11973, USA

[3]School of Materials Science and Engineering, Georgia Institute of Technology, Atlanta, GA 30332, USA

[4]Department of Chemistry, Georgia State University, Atlanta, GA 30302, USA

[#] G.H. and L.L. contributed equally to this work.

*Corresponding authors, E-mails: (G.H.) gxh5312@psu.edu; (R.J.) rbj5326@psu.edu

I. Chemicals and Methods

Chemicals

Tetrachloroauric (III) acid ($HAuCl_4 \cdot 3H_2O$, 99.999% metal basis, Aldrich), phenylethyl mercaptan (PET, >99%, Aldrich), sodium borohydride (powder, ≥98%, Sigma), benzimidazole (98%, Aldrich), 2-bromopropane (99%, Aldrich), dimethyl sulfide (SMe_2, >99%, Aldrich), potassium carbonate (K_2CO_3, 99%, Aldrich), Toluene-d8 (99 atom % D, Sigma), methanol, ethanol, ethyl ether, ethyl acetate, pentane, dichloromethane (DCM), chloroform, *n*-hexane and acetonitrile (HPLC grade for all solvents) were used as received. Thin-layer chromatography (TLC) plates were from iChromatography (silica gel, 250 μm).

Synthesis of chloro(dimethylsulfide)gold(I) (AuCl(SMe_2))

$HAuCl_4 \cdot 3H_2O$ (500 mg, 1.27 mmol) was dissolved in ethanol (20 mL), followed by the addition of SMe_2 (280 μL, 3.81 mmol), and the solution was vigorously stirred for 2 hours. After that, the white precipitate was collected by centrifugation, which was washed with ethyl ether and finally dried to give the product as a white powder.

Synthesis of 1,3-diisopropylbenzimidazolium bromide (iPr$_2$-bimy·HBr)

Benzimidazole (1.18 g, 10 mmol) and K_2CO_3 (760 mg, 5.5 mmol) were added into acetonitrile (8 mL) and the mixture was rapidly stirred at ambient temperature for 1 hour. Following that, 2-bromopropane (5.4 mL, 57.5 mmol) was added to the suspension, and the reaction mixture was vigorously stirred under reflux conditions for 24 hours, followed by the addition of a second portion of 2-bromopropane (5.4 mL, 57.5 mmol). The reaction mixture was vigorously stirred under reflux for an additional 48 hours. After removing the solvent under reduced pressure, DCM was added to the residues, and the upper supernatant after centrifugation was collected. The solvent of the supernatant was removed under reduced pressure to produce a spongy solid, which was washed by ethyl acetate to afford the desired product as a white powder.

Synthesis of NHC-Au-Br complex ($^{i}Pr_2$-bimy·AuBr)

$^{i}Pr_2$-bimy·HBr (1337.4 mg, 4.725 mmol), AuCl(SMe_2) (1393.4 mg, 4.725 mmol), and K_2CO_3 (653.5 mg, 4.725 mmol) were added into acetone (20 mL) and the mixture was vigorously stirred under reflux conditions for 2 hours. After that, the solvent in the suspension was removed under reduced pressure. DCM was added to the residues, and the upper supernatant after centrifugation was collected. The solvent of the supernatant was removed under reduced pressure to give the solid product, which was washed with pentane and finally dried to afford the desired product as a gray powder.

Synthesis of Au_{42} quantum rods

$^{i}Pr_2$-bimy·AuBr (120 mg, 0.25 mmol) and PET (67 μL, 0.5 mmol) were dissolved in a mixture of chloroform (15 mL) and ethanol (5 mL). The mixture gradually turned cloudy white (Au^{I}-PET formed). After 1 hour of stirring, the suspension was reduced to nanoclusters (NCs) by the addition of sodium borohydride (95 mg, 2.5 mmol, dissolved in 5 mL ice-cold H_2O) under darkness. A black solution was formed. The reaction continued for 6 hours, and then the solvent was removed under reduced pressure. The mixture of Au NCs was thoroughly washed with methanol, extracted with DCM, and concentrated for TLC separation. The mixture of Au NCs was pipetted on a TLC plate, and the separation was conducted in a developing tank (developing solvent 1:1 (v/v) DCM:n-hexane) (Figure S1). The band corresponding to $Au_{42}(PET)_{32}$ was cut off and dissolved in DCM for characterization. The optical measurements were performed in toluene or 2-methyltetrahydrofuran (2-MeTHF).

Characterization

UV-Vis-NIR spectra were collected on a UV-3600 Plus UV-Vis-NIR spectrophotometer (Shimadzu). Steady-state photoluminescence spectra were measured on a FLS-1000 spectrofluorometer (Edinburgh). Near infrared PL was measured using a wide-range photomultiplier tube (PMT-1700, 500 - 1700 nm) cooled to -80 °C by liquid nitrogen. The PL lifetimes excited at 450 nm were measured by time-correlated single photon counting (TCSPC) on the same instrument.

The absolute PLQY of Au_{42} excited at 808 nm was measured by using an integrating sphere ($\Phi_R = 18\%$). The PLQY (Φ_S) at other excitation wavelengths were determined by a relative method (i.e. comparing to $\Phi_R = 18\%$) under the same instrument settings. The calculations used the following equation:

$$\Phi_S = \Phi_R \left(\frac{I_S}{I_R}\right)\left(\frac{1-10^{-A_R}}{1-10^{-A_S}}\right)\left(\frac{n_S}{n_R}\right)^2$$

where, Φ_R is the PLQY of the reference standard (18% for Au_{42} excited at 808 nm), I is the integrated PL intensity (S or R), A is the optical density of solution at various excitation wavelengths (S or R), and n is the refractive index of the solvent.

Transient absorption measurements were carried out using a broadband pump-probe setup, which is pumped by a 1 kHz Ti:Sapphire laser system (Spitfire, Spectra-Physics). The pump wavelengths were generated by a commercial optical parametric amplifier (TOPAS-C, Light Conversion). For femtosecond TA measurements, the supercontinuum probe light is generated by focusing the fundamental pulse (800 nm) into a sapphire plate. The probe light is split into signal and reference beams. The pump-probe delay was controlled by a mechanical delay line. For nanosecond TA measurements, the probe light is generated with a fiber laser (Leukos), and the delay times up to microseconds are controlled by an electronic delay configuration. TA measurements were performed in toluene, and the optical density of solution was adjusted to ~0.2 OD (2 mm cuvette) at excitation wavelength of 800 nm. The polarization of pump and probe pulse was set to magic angle (54.7°) to measure the isotropy signal. The transient absorption spectra were further analyzed using the publicly available program Glotaran based on the statistical fitting package TIMP.[s1,s2] A branched target model was performed for the fs TA data, while a sequential model was performed for ns TA data.

II. Supporting Figures (S1-S14) and Table (S1):

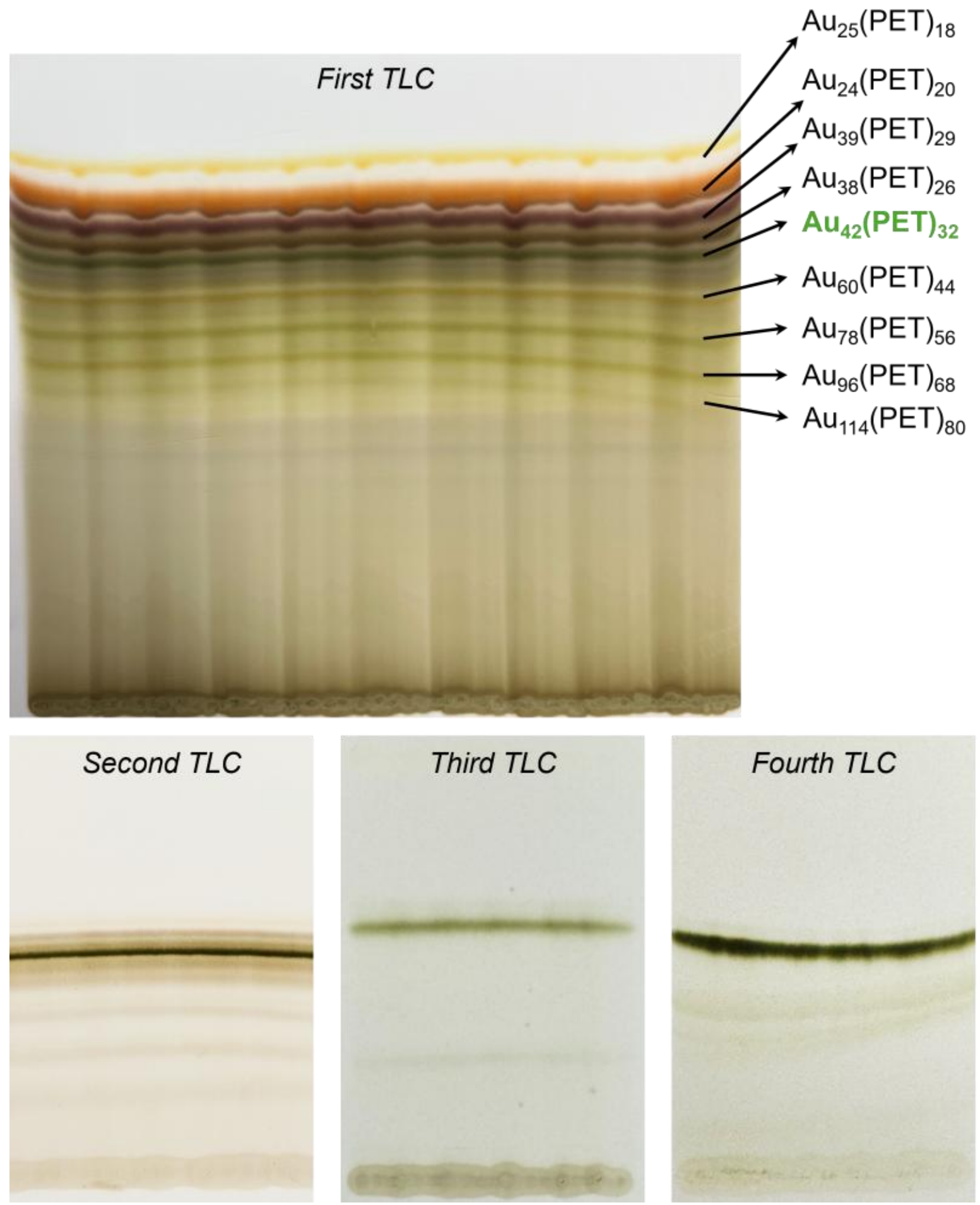

Figure S1. TLC separation of $Au_{42}(PET)_{32}$ from the product mixture (top) and multiple runs (bottom). The 4th TLC purified sample was used in spectroscopic measurements.

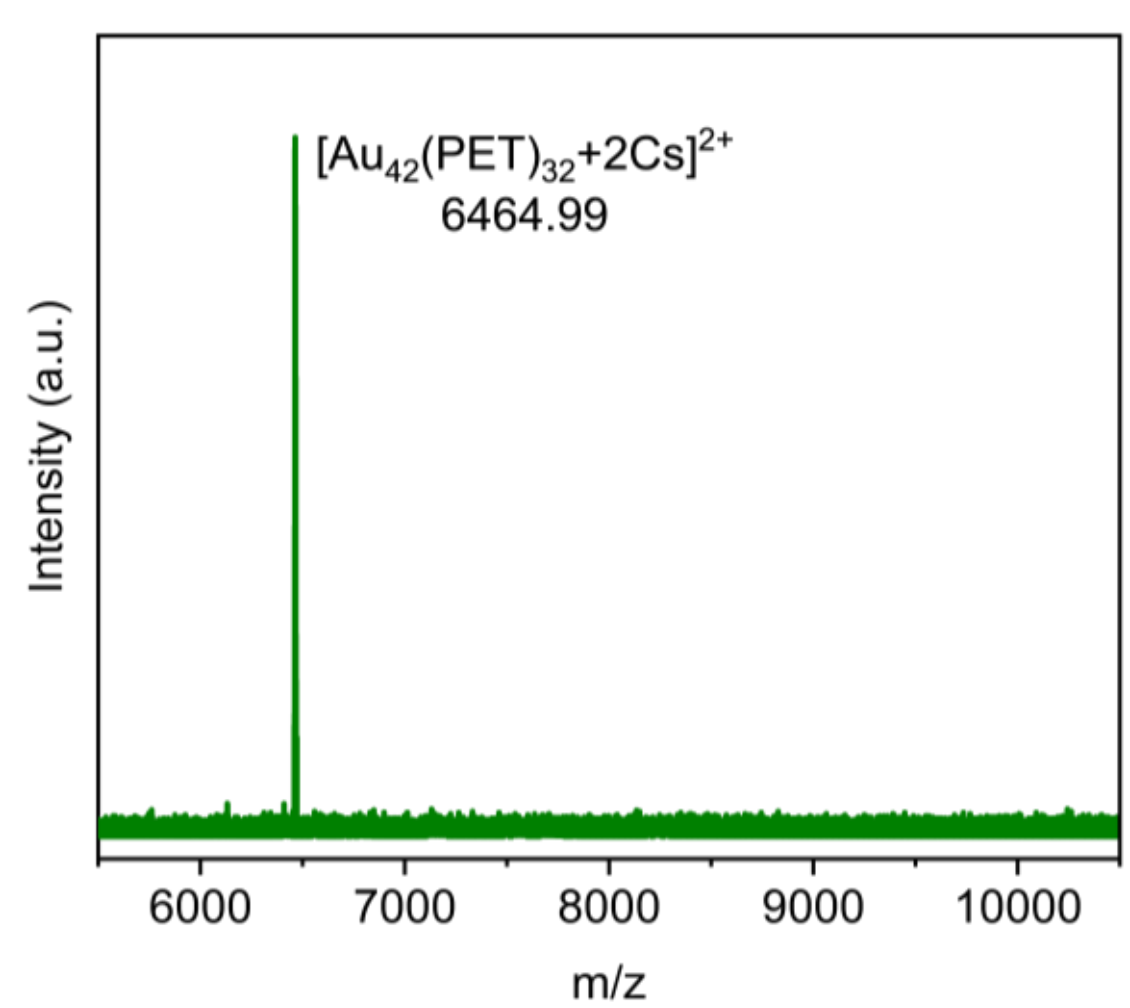

Figure S2. ESI mass spectrum of $Au_{42}(PET)_{32}$, which was after the 4th TLC run. Cesium acetate was added during ESI to impart charges to the nanocluster by forming an adduct.

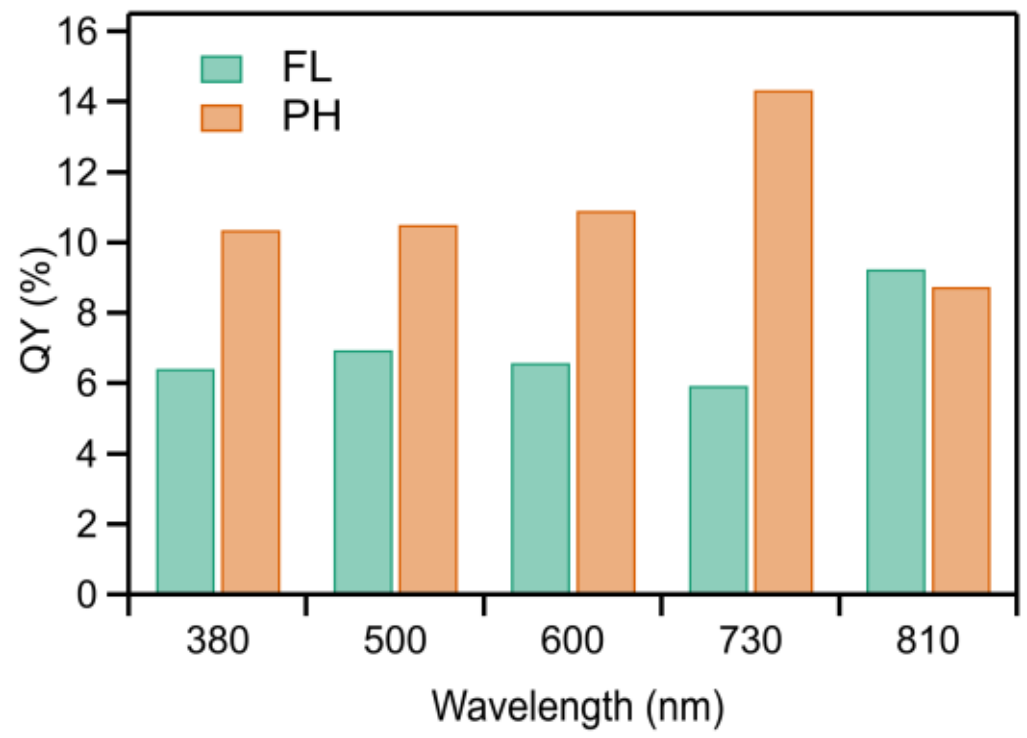

Figure S3. FL and PH quantum yields at different excitation wavelengths.

Table S1. Parameters of cryogenic PL properties of Au_{42} in deaerated 2-MeTHF.

Temperature (K)	Total QY (%)[1]	QY of FL (%)	QY of PH (%)	τ of S_1 (ns)[2]	k_r of S_1 (× 10^8 s^{-1})	k_{nr} of S_1 (× 10^8 s^{-1})
298	18.1	10.1	8.0	0.64	1.58	14.0
260	21.3	11.7	9.6	0.76	1.54	11.6
220	24.9	13.5	11.4	0.93	1.45	9.30
180	28.0	13.3	14.6	1.10	1.21	7.88
140	33.3	16.1	17.2	1.12	1.44	7.49
100	42.6	17.9	24.7	1.14	1.57	7.20
80	45.9	19.8	26.1	1.20	1.65	6.68

Notes: [1]QY were measured upon excitation at 806 nm.
[2]FL lifetimes were measured excited at 450 nm.

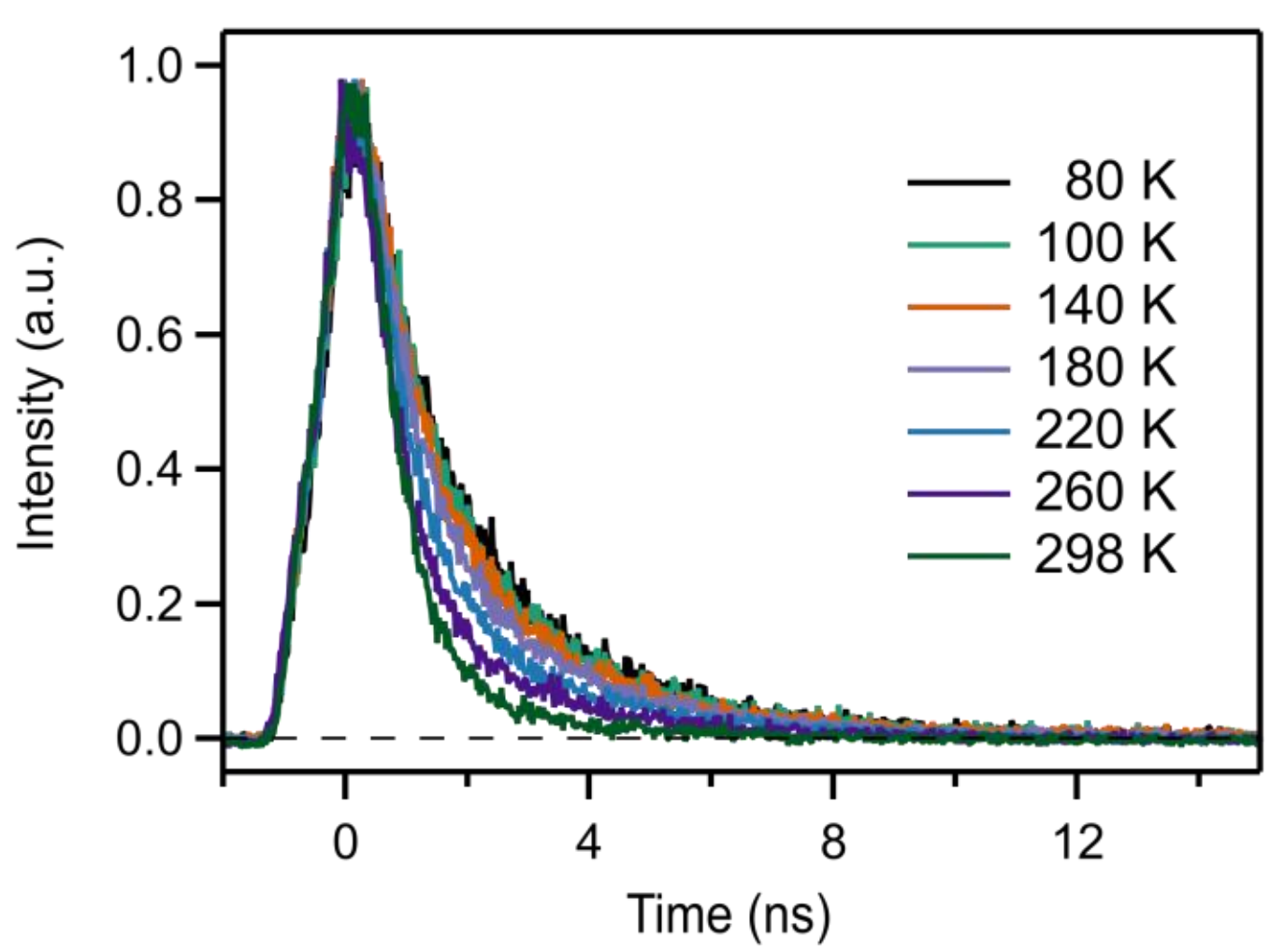

Figure S4. FL kinetics of Au_{42} at different temperatures.

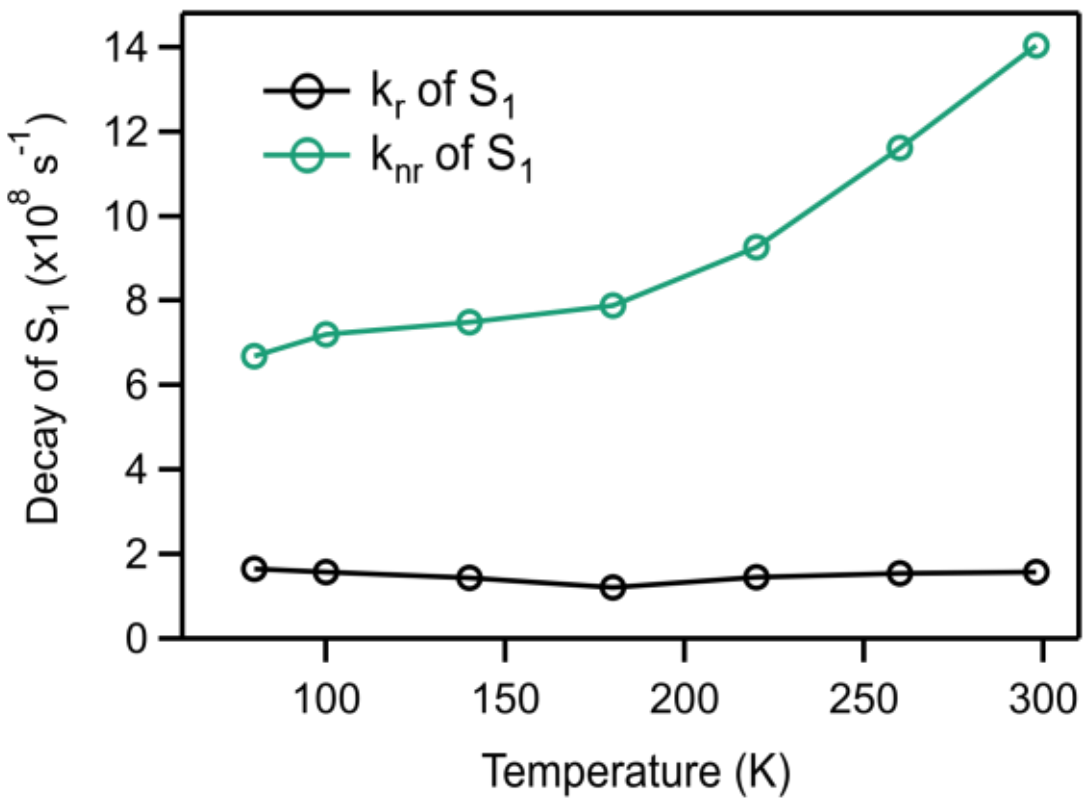

Figure S5. Radiative and nonradiative rate constants of the S_1 state for Au_{42}.

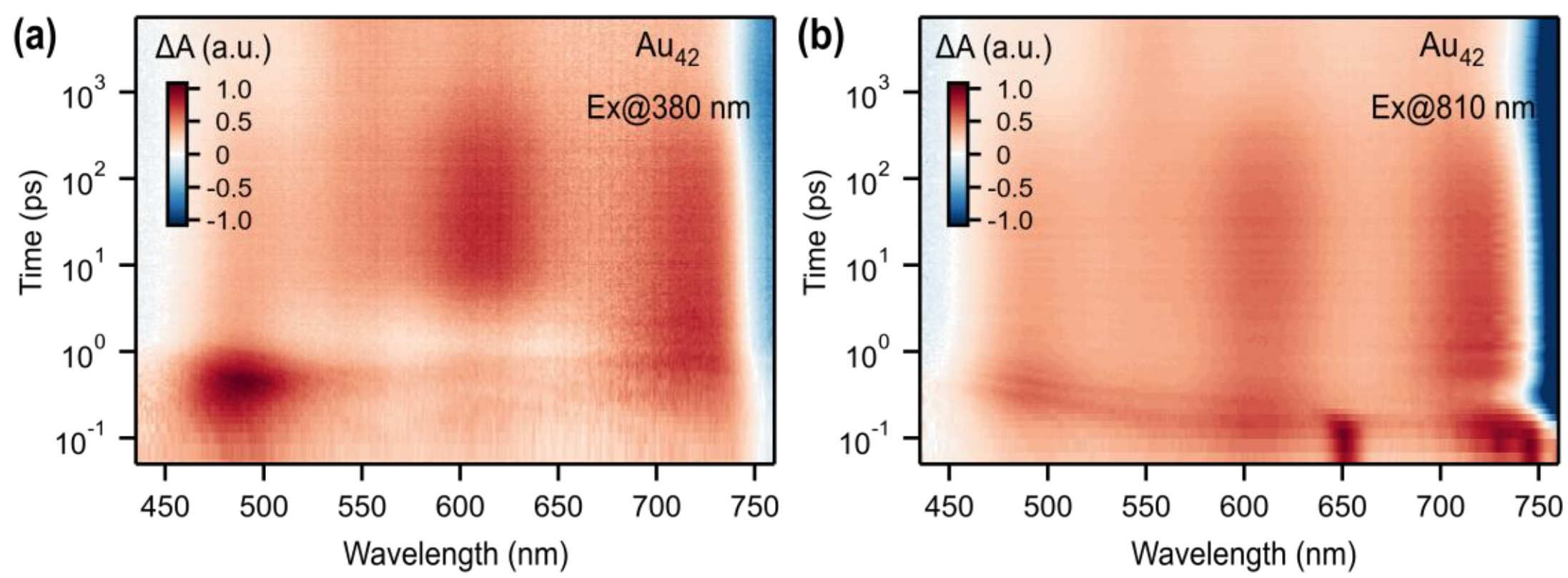

Figure S6. Transient absorption data maps in visible region of Au_{42} excited at 380 nm (a) and 810 nm (b).

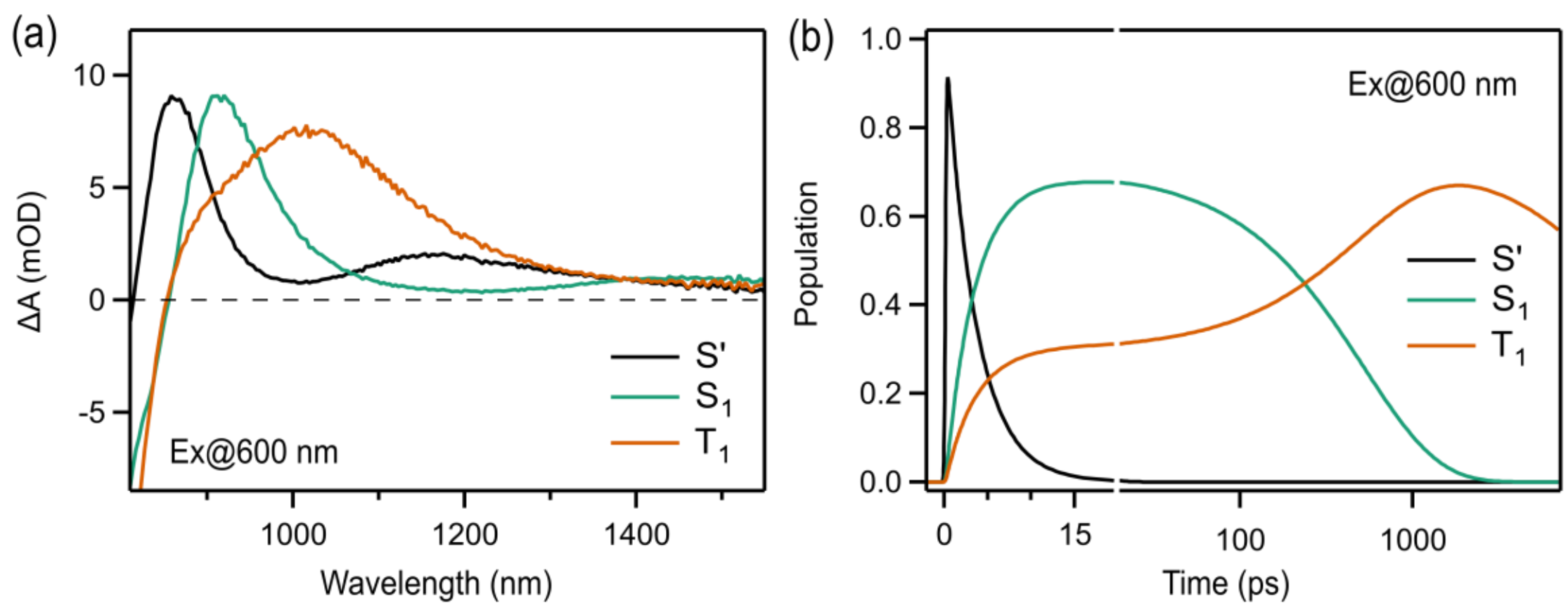

Figure S7. Target analysis based on a branched model for TA data of Au_{42} at excitation of 600 nm. Species associate spectra (a) and population evolution (b).

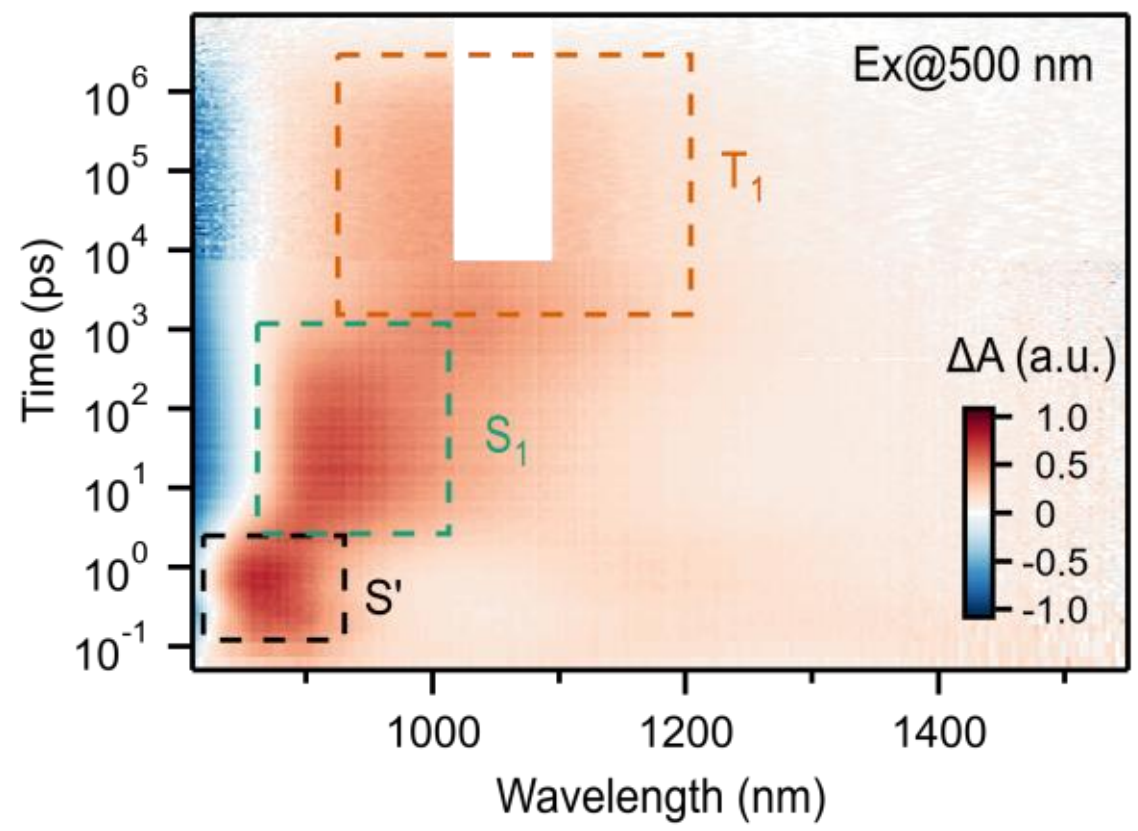

Figure S8. Transient absorption data map of Au_{42} in toluene at excitation of 500 nm. Note: the white box for ns TA is the removal of residual pump-laser peak of the probe.

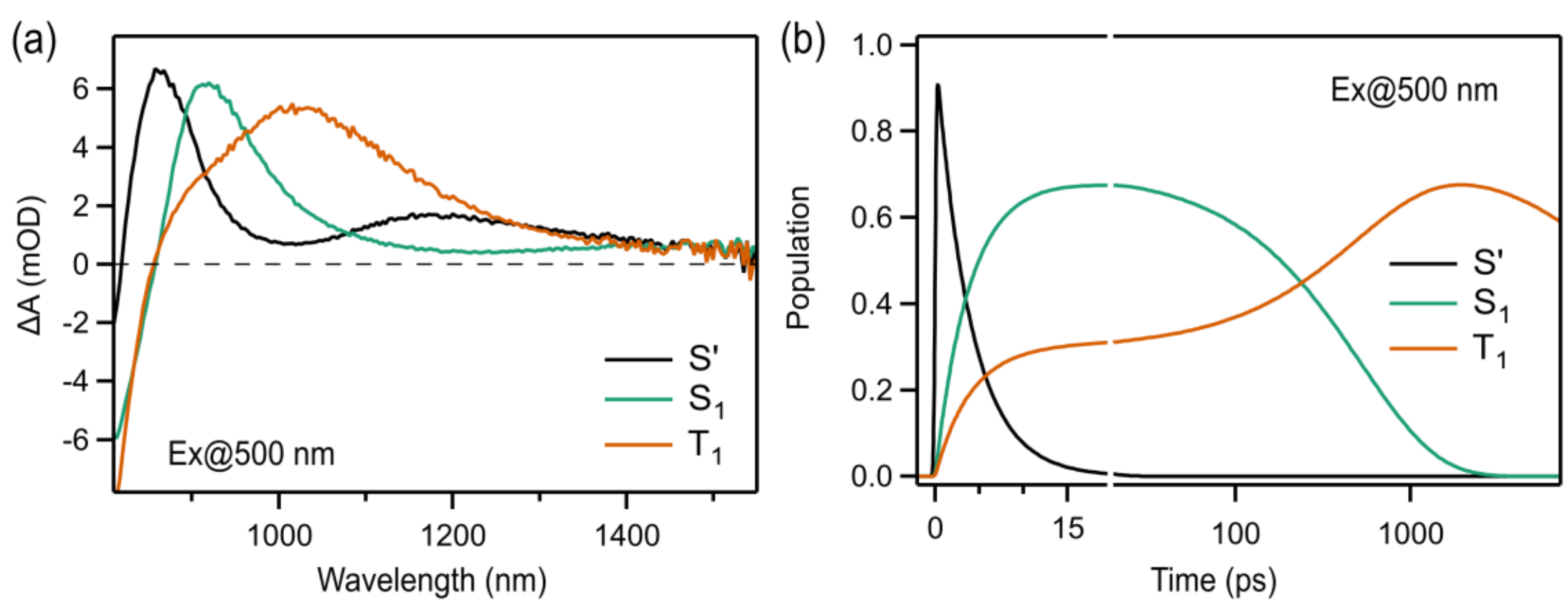

Figure S9. Target analysis based on a branched model for TA data of Au_{42} at excitation of 500 nm. Species associate spectra (a) and population evolution (b).

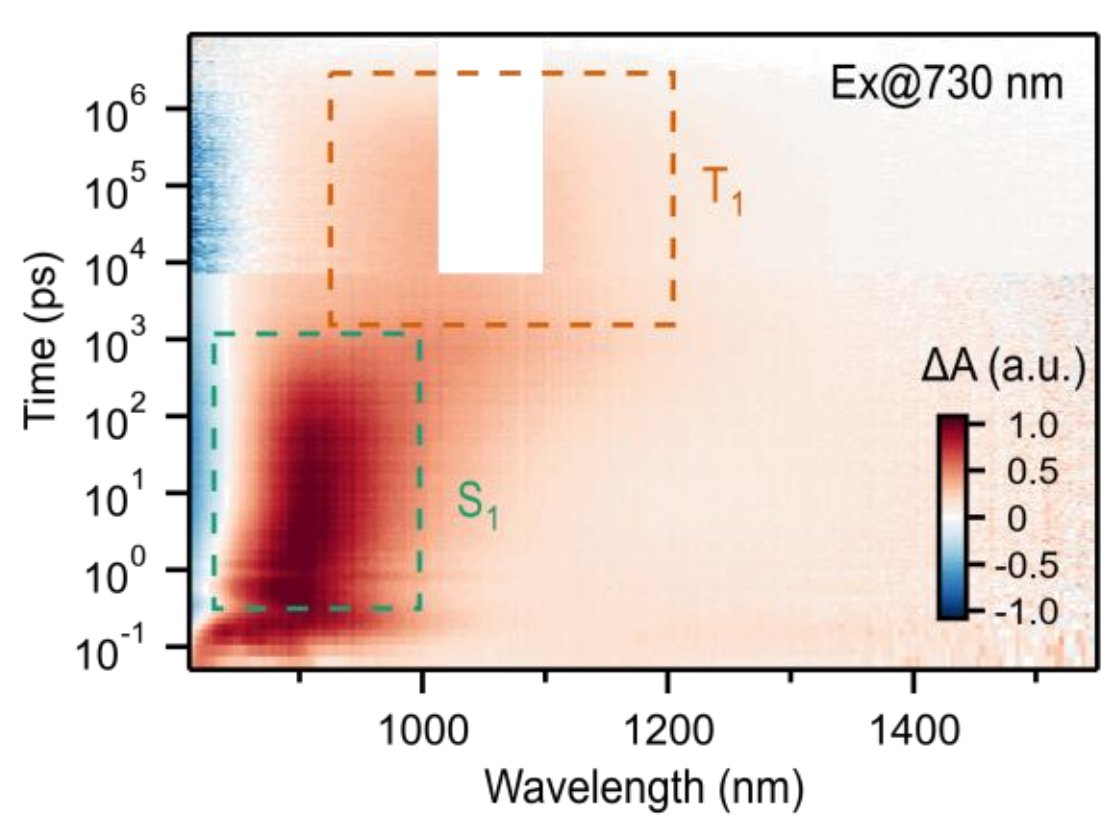

Figure S10. Transient absorption data map of Au_{42} in toluene at excitation of 730 nm. Note: the white box for ns TA is the removal of residual pump-laser peak of the probe.

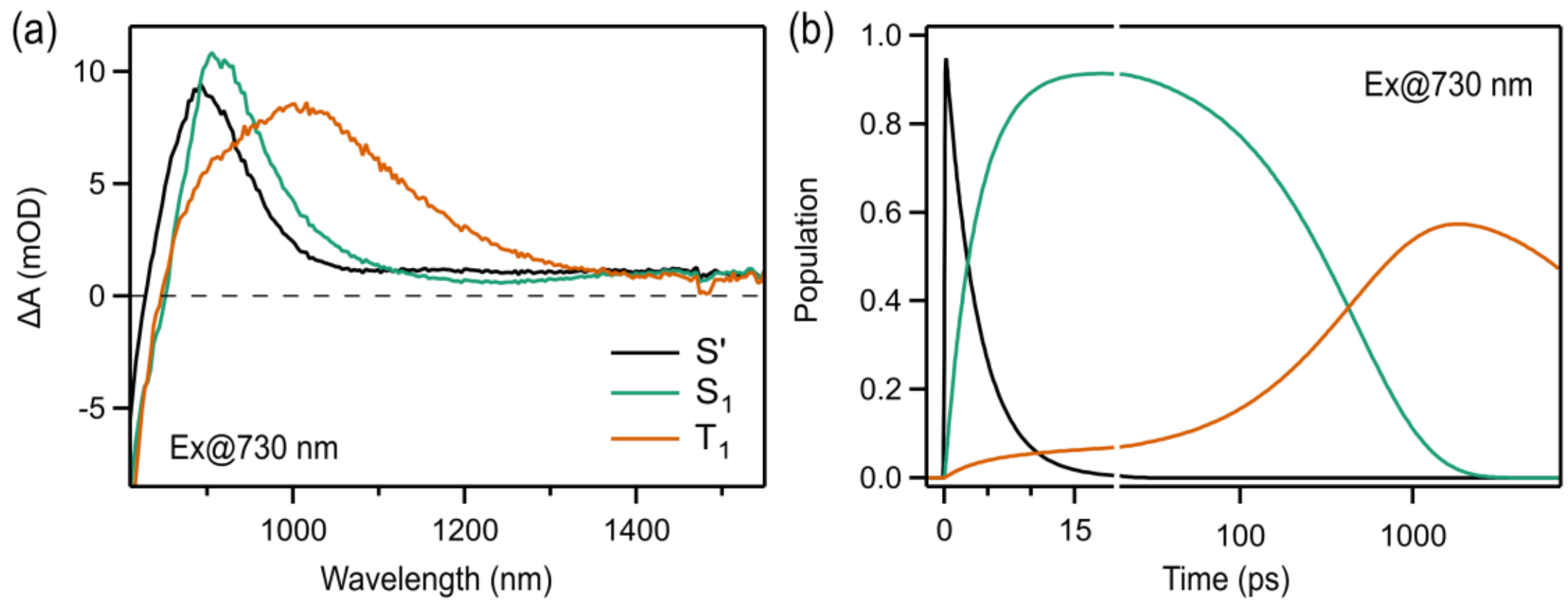

Figure S11. Target analysis based on branched model for TA data of Au_{42} at excitation of 730 nm. Species associate spectra (a) and population evolution (b).

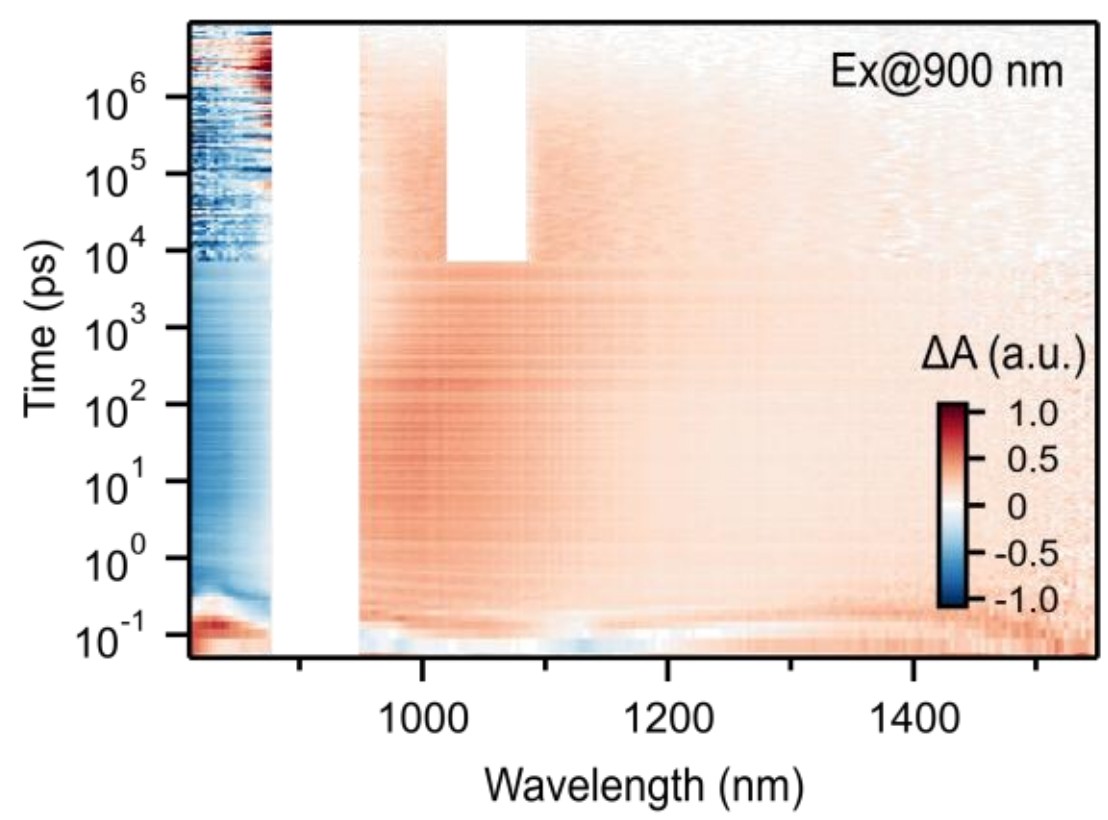

Figure S12. Transient absorption data map of Au_{42} in toluene at excitation of 900 nm. Note: the white box for ns TA is the removal of residual pump-laser peak of the probe.

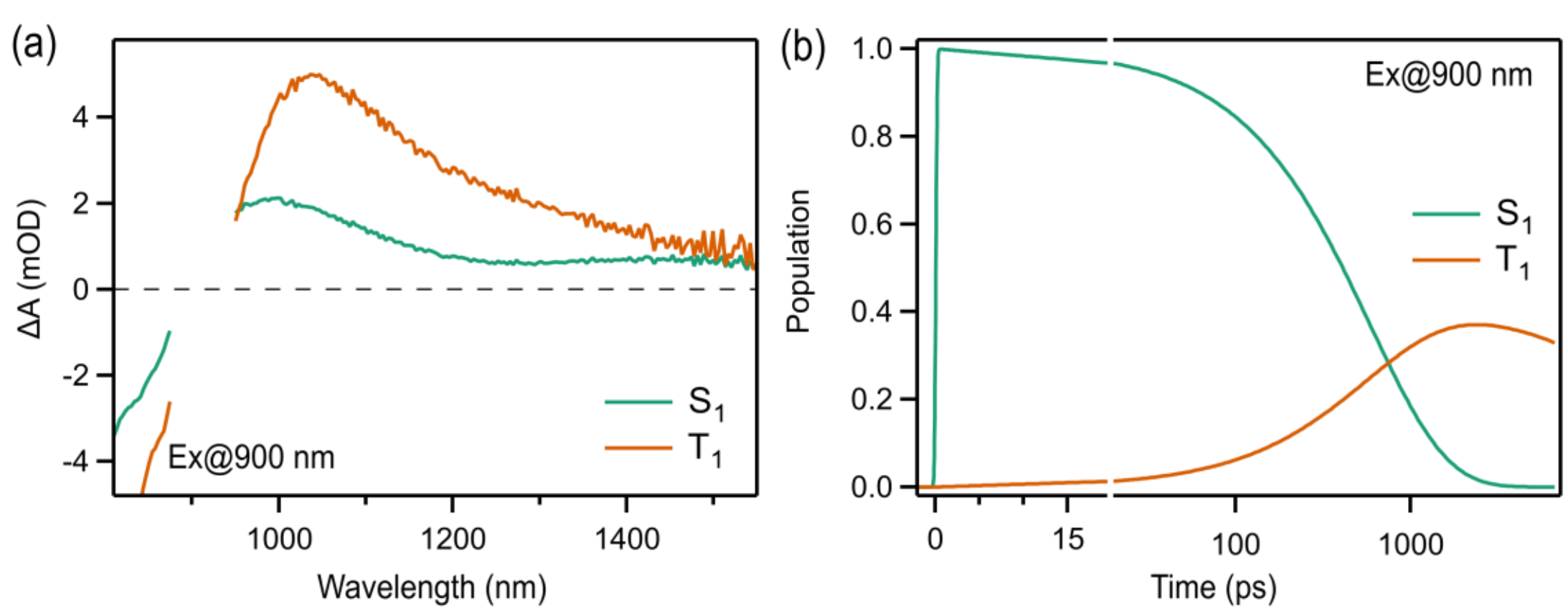

Figure S13. Target analysis based on branched model for TA data of Au_{42} at excitation of 900 nm. Species associate spectra (a) and population evolution (b).

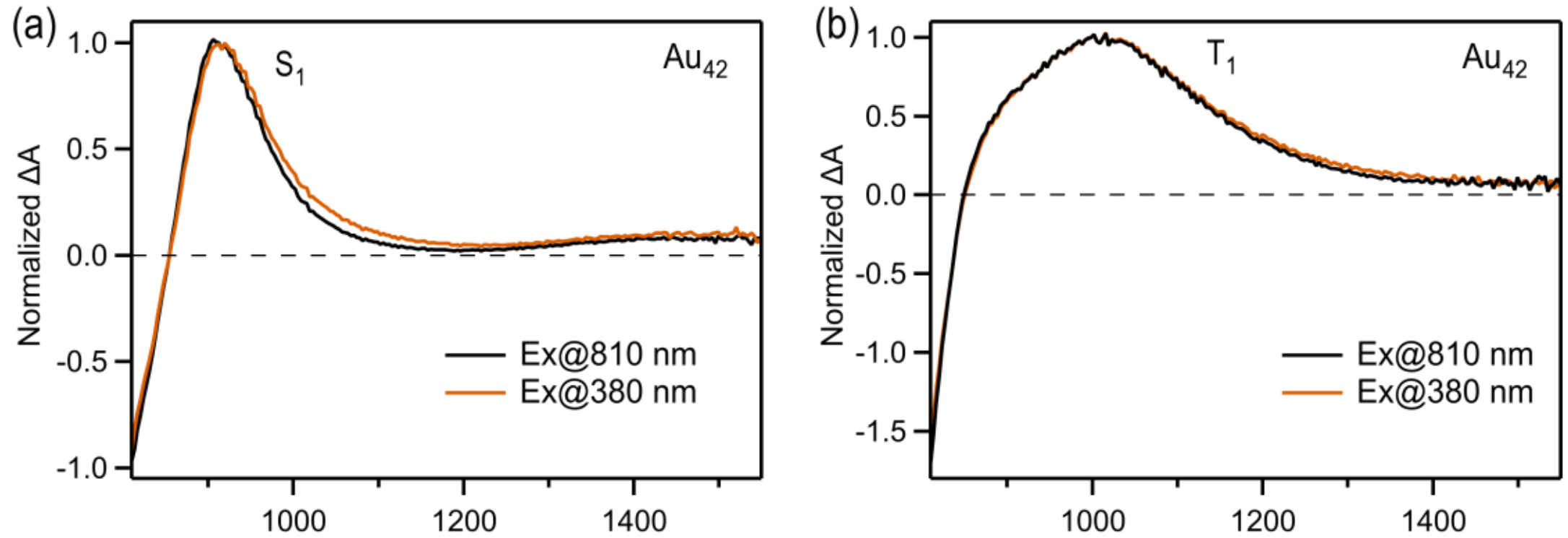

Figure S14. Spectral comparison of S_1 (left) and T_1 (right) at excitation of S_1 (810 nm) and S_n (380 nm).

Supporting References:

(s1) Snellenburg, J. J.; Laptenok, S. P.; Seger, R.; Mullen, K. M.; Stokkum, I. H. M. van. Glotaran : A Java-Based Graphical User Interface for the R Package TIMP. *J. Stat. Soft.* **2012**, *49* (3), 1–22.

(s2) van Stokkum, I. H. M.; Larsen, D. S.; van Grondelle, R. Global and Target Analysis of Time-Resolved Spectra. *Biochim. Biophys. Acta - Bioenerg.* **2004**, *1657* (2–3), 82–104.
